# Prognostic and Predictive Cross-Roads of Microsatellite Instability and Immune Response to Colon Cancer

**DOI:** 10.3390/ijms21249680

**Published:** 2020-12-18

**Authors:** Luigi Laghi, Francesca Negri, Federica Gaiani, Tommaso Cavalleri, Fabio Grizzi, Gian Luigi de’ Angelis, Alberto Malesci

**Affiliations:** 1 Department of Medicine and Surgery, University of Parma, 43121 Parma, Italy; federica.gaiani@unipr.it (F.G.); gianluigi.deangelis@unipr.it (G.L.d.A.); 2 Laboratory of Molecular Gastroenterology, Humanitas Clinical and Research Center IRCCS, Rozzano, 20089 Milan, Italy; tommaso.cavalleri@humanitasresearch.it; 3 Medical Oncology Unit, University Hospital of Parma, 43121 Parma, Italy; fnegri@ao.pr.it; 4 Department of Immunology and Inflammation, Humanitas Clinical and Research Center IRCCS, Rozzano, 20089 Milan, Italy; fabio.grizzi@humanitasresearch.it; 5 Department of Biomedical Sciences, Humanitas University, Pieve Emanuele, 20090 Milan, Italy; alberto.malesci@hunimed.eu; 6 Department of Gastroenterology, Humanitas Clinical and Research Center IRCCS, Rozzano, 20089 Milan, Italy

**Keywords:** tumor-infiltrating lymphocytes (TILs), microsatellite instability (MSI), adjuvant therapy, prognostic and predictive value, colon cancer

## Abstract

Understanding molecular features of colon cancer has shed light on its pathogenesis and progression. Over time, some of these features acquired clinical dignity and were incorporated in decision making. Namely, microsatellite instability (MSI) due to mismatch repair of defects, which primarily was adopted for the diagnosis of Lynch syndrome, became recognized as the biomarker of a different disease type, showing a less aggressive behavior. MSI tumors harbor high amounts of tumor infiltrating lymphocytes (TILs) due to their peculiar load in neoantigens. However, microsatellite stable colon cancer may also show high amounts of TILs, and this feature is as well associated with better outcomes. High TIL loads are in general associated with a favorable prognosis, especially in stage II colon cancer, and therein identifies a patient subset with the lowest probability of relapse. With respect to post-surgical adjuvant treatment, particularly in stage III, TILs predictive ability seems to weaken along with the progression of the disease, being less evident in high risk patients. Moving from cohort studies to the analysis of a series from clinical trials contributed to increase the robustness of TILs as a biomarker. The employment of high TIL densities as an indicator of good prognosis in early-stage colon cancers is strongly advisable, while in late-stage colon cancers the employment as an indicator of good responsiveness to post-surgical therapy requires refinement. It remains to be clarified whether TILs could help in identifying those patients with node-positive cancers to whom adjuvant treatment could be spared, at least in low-risk groups as defined by the TNM staging system.

## 1. The Journey of Adaptive Immune Response to Colon Cancer from Translational to Clinical Research

### 1.1. Transition of Biomarkers to the Clinical Ground alongside the TNM Staging System

In treating colon cancer, the pillar for prognostication and prescription of adjuvant therapy after surgery remains the TNM system [1,2,3,4,5,6,7,8,9,10,11]. However, in recent years, several biological features of the disease have proven pivotal for predicting post-surgical behavior of colon cancer and guiding drug prescription in the clinical ground [4,5,6]. Acknowledged examples include sparing of adjuvant therapy in patients with stage II colon cancer showing microsatellite instability (MSI) [7,8], effectiveness of biological therapy according to RAS-pathway status in advanced disease [9] prescription of immune therapy in patients with advanced MSI colon cancer, and possibly the responsiveness of the same tumors to kinase inhibitors if showing hypermethylation of *MLH1* promoter and *RAS/BRAF* wild-type [10]. This list may soon include the density of tumor infiltrating lymphocytes (TILs), which partially overlaps and shares similarities with MSI as to patient outcome. Similarly as for other biomarkers, the proper interpretation of these parameters requires considering their relationship with tumor stage at diagnosis, to exploit the information concerning the postsurgical outcome of patients, which may envision or not the administration of adjuvant therapy [1,2,3,4,5,6,7,8,9,10,11,12,13,14,15]. Accordingly, TIL density may have both prognostic and predictive value, and the parallelism with MSI is enlightening.

### 1.2. Colorectal Cancers with MSI as a Different Disease Subtype

The detection of MSI, due to DNA mismatch repair (MMR) defects as currently assessed by immune-histochemistry, was first proposed as the method of choice for the molecular screening aimed at identifying the patients with Lynch syndrome [11,16,17]. Over time, MSI progressively emerged as the biomarker of a disease subtype marked by a low metastatic potential. This notion stemmed from the stage of distribution at diagnosis of MSI colon cancers, more frequently detected at an early stage (i.e., mainly stage II), conveying the idea of a mild, slow-progressing disease [18]. Along this view, universal MSI screening, allows in parallel to reach the systematic search for Lynch syndrome patients and the proper allocation to post-surgical treatment for approximately 15% of colon cancer patients.

### 1.3. Host Adaptive Immune Response as Predictor of Colorectal Cancer Outcome

**Figure 1:**
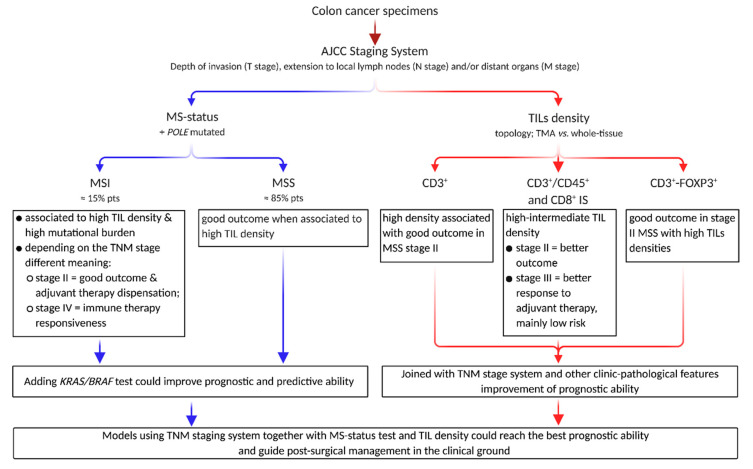
The prognostic and predictive ability of pathological and biological colon cancer features interact to impact post-surgical outcome. In addition to the pathological AJCC cancer staging system, the post-surgical medical decisions are implemented by the MS-status assessment, plus mutation in the RAS family and POLE gene. TILs progressively became relevant players in forecasting patient outcome in different clinical settings, possibly including the responsiveness adjuvant treatment. Abbreviations: AJCC, American Joint Committee on Cancer; IS, Immunoscore; MS-status, microsatellite status; MSI, microsatellite instability; MSS, microsatellite stability; TIL, tumor-infiltrating lymphocyte; TMA, tissue microarray.

Long before MSI, the amount of TILs surrounding colon cancer emerged as a host feature which allowed stratifying the outcome of colon cancers [19]. The first systematic report by Jass concerned rectal cancers and proposed this characteristic, by then directly assessed by optical microscopy, as an independent prognostic factor (Figure 1) [20]. It should be noted that the acronym TIL has been largely adopted, although several types of lymphocytic reactions can be identified in the tumor microenvironment. In fact, pathologists have been able to recognize up to four components of lymphocytic reaction with respect to their location; namely Crohn’s like, peritumoral, intratumoral and TIL, as identified by locations with respect to cancer cells [21]. Nevertheless, the acronym TIL is widely employed, possibly followed by the specification of their location. At a later time, in a contemporary molecular era, a comprehensive expression analysis of colon cancer identified the immune reaction to colon cancer and therein the density of TILs as a primary prognostic feature [12,22]. The repechage of TILs prognostic value originated since then an array of papers that progressively confirmed and refined such value. Definitely, TILs are a major prognostic factor in early (i.e., stage I and II) colon cancers, and a matter of ongoing investigation in later stages, with current emphasis on stage III (Figure 1) [13].

Several factors influence the quantification of TILs, comprising their subtypes (as assessed by clusters of differentiation), locations (at the tumor front vs intra-tumoral or in the whole tissue specimen), and measurements methods (from tissue microarrays (TMA) to whole tissue slides) that now are incorporating artificial intelligence [14,15,23,24]. In any event, irrespective of the above-mentioned inherent differences among the studies, several TIL subpopulations have been recognized as determinants of colon cancer outcome in the last fifteen years.

### 1.4. TILs as Host Response to Hypermutated Cancers Triggered by Neoantigen Production

However, before the analysis of TILs became a widely explored field, it was recognized that MSI colon cancers harbor on average significantly higher loads of TILs than MSS colon cancers [14,25,26,27,28,29,30]. The contribution of this feature to their lower metastatic potential was not analyzed in large series, although it was put forward as a remarkable feature. Indisputably, high TIL loads of MSI colon cancer could be attributed to the huge amount of tumor neoantigens arising in MSI colon cancer, due to un-repaired frameshift mutations that lead to the synthesis of truncated proteins [31,32,33,34]. The high immunogenicity may not be the only explanation for better clinical behavior of MSI colon cancers, which could also be justified by the same occurrence of unrepaired random mutations [35]. Some of these may be detrimental to tumor evolution and not subject to clonal selection, lacking selective advantages, and may rather result in clonal dead ends. In the current era of next generation sequencing, the difference in the amount of mutations between MSI and MSS colon cancer is better measured as tumor mutational burden and differs by thousands of units upon the MS-status of the tumors [36,37,38]. Inherently unstable MSI cancers are not uniquely associated with high mutational burden, an occurrence which is detectable also in ultramutated colon cancer identified by pathogenic somatic mutations in the *POLE* proofreading domain. This molecular subset of colon cancers shows, as well, increased TIL loads, although this type of damage occurs in a small (approximately 1%) subgroup of patients (see below) [39]. Thus, subgroups of colon cancers with different molecular pathogenesis have significantly different TIL loads. In any event, although the contribution of MSI colon cancers to the total prognostic impact of TILs is relevant, it does not explain it all [40]. In fact, the TIL amount remains a significant prognostic factor in MSS colon cancers even once MSI tumors are excluded [14]. This is safely stated as to stage II, while some uncertainties remain as to stage III, in which the scenario of patient outcome would be incomplete without considering the administration of adjuvant therapy. As of now, MSI is an acknowledged prognostic molecular trait in stage II colon cancer, which implies therapeutic decision, that spares adjuvant chemotherapy to patients with MMR deficient cancers diagnosed in this stage. This role originated from the joined evidence of the better outcome of MSI colon cancer joined to the lack of benefit from adjuvant therapy in patients with MSI colon cancer in comparison to patients with stage II MSS tumors [6,7]. Such a role is not yet acknowledged to TILs, and their high amount has rather been advocated to identify patients who may better respond to chemotherapy [41,42].

### 1.5. Heterogeneity of TILs and Their Assessment

Methodological differences have been largely overcome in determining the MS and/or MMR-status of colon cancer. In general, MMR defects determination by immunohistochemistry is now routinely performed in pathology departments, and the results are overlapping with those obtained by molecular MS-status assessment. For TILs assessment, while a true standard is lacking, the results may depend upon the type of sub-population, and there is a variety of differences in the measurement methods [43,44,45]. Notwithstanding, the results are anyway concordant in supporting a direct relationship between the TIL amount and better outcomes. Different subpopulations have been assessed over time, chiefly CD45RO^+^, CD3^+^, CD8^+^, and forkhead box P3 (FOXP3^+^) cells (see below). A relevant degree of overlap exists for the prognostic meaning of these TIL populations. While efforts have been directed at identifying the “best” TIL marker, this remains undetermined (and somehow contradictory) as data progressively pointed at coupling two TIL populations and building up related scores to determine the magnitude of lymphocyte infiltration [46]. Locations of TILs with respect to tumor topography has been also variably explored, and eventually coupling two locations has been employed in translational studies, obtaining measurements at the invasion margin and at the center of the tumor [12]. Although certain markers were not independent at multivariable analysis in some studies (see below), their double measurement at dual locations may lead to a more precise estimate of the infiltration. However, methodological and technical improvements over time progressively allowed analyzing an increasingly large portion of tumor tissue, thus moving from TMA to whole tissue slides.

### 1.6. Heterogeneity of TILs and Patient Outcome, with Focus on Stage II-III CRC

Starting from the paper published in the New England Journal of Medicine [22], the research duo composed of Pages and Galon introduced the notion that the absence of vascular, lymphatic, and perineural invasion in colon cancer was associated with increased survival, as well as with increased infiltrating immune cells and mRNA levels for products of type 1 helper effector T cells. The tumors with such features harbored an increased number of CD8^+^ T cells, including early memory and effector T cells, and the association of such coordinated immune response with better outcome could be summarized by a high density of CD45RO^+^ cells as assessed by immunohistochemistry. Shortly after, they showed in one other landmark paper that type, density, and location of immune cells within human colon cancers can predict clinical outcome [12]. This paper first showed that the density of CD3^+^ cells measured both at the invasive margin and the tumor core provides an excellent prognostication, competing with standard histopathological staging parameters. As a matter of fact, these two papers paved the way to the notion that the measurement of a coordinated adaptive immune response in tumor tissue could be evaluated by immune-histochemistry and serve as a prognostic tool. The coupling of two markers (namely CD45RO and CD8) at two locations from patients (*n* = 602) with early-stage tumors (i.e., stage I and II) refined the measurement approach, leading to building five prognostic strata, the best outcome being achieved by those patients having high densities of both markers in their tumors [47]. Namely, only 5% of patients with high-high CD45RO^+^ and CD8^+^ TILs in their colon cancer experienced recurrence, as compared to 75% of relapses among those patients whose tumors harbored low-low densities of these TILs. Eventually, such measurement, referred to as the Immunoscore (IS), has been inversely associated with tumor progression across stages, and able to provide an excellent prediction of survival [13]. The mechanistic implications of the immune response synthetized by a simple immune-histochemical score would reflect the predominant type of responses elicited by tumors, predominant Th1-type being associated with good outcome as opposed to predominant Th17 being associated with a worse one [48]. Most studies were managed on TMA, but later the assessment by CD3^+^ and CD8^+^ TILs landed on whole tissue sections within the international study for the validation of a consensus IS launched in 2012 [49,50]. Meanwhile, the prognostic equivalence between CD8^+^/CD45RO^+^ and CD3^+^/CD8^+^ IS was reported in the context of colon cancer molecular typing [40].

Meanwhile, the relationships between the individual prognostic value of the densities of CD45RO^+^, CD8^+^, and FOXP3^+^ TILs and patient outcome have been evaluated by Salama et al. in TMA from 967 stage II and III colon cancers [51]. In their work, they appraised that FOXP3^+^ TILs were the only prognostic indicator independent from stage and vascular invasion, and that high densities of FOXP3^+^ cells in the tumor remained positively associated with better outcome in stage II patients, independently from perineural and vascular invasion (while an opposite behavior emerged when measured in normal tissue) at multivariable modeling. One other direct comparison, companion to a meta-analysis, was published in 2010 by Nosho et al., who compared the prognostic power of CD45RO^+^, CD3^+^, CD8^+^, and FOXP3^+^ cells in neoplastic epithelial areas of 768 TMA from colon cancer patients, encompassing all stages [52]. In their regression model CD45RO^+^, CD8^+^, and FOXP3^+^ were associated with longer cancer specific survival based on univariable analysis, but only the density of CD45RO^+^ TILs retained a significant association when applying multivariable analysis. Notably, MSI and *LINE-1* hypermethylation were independently associated with the density of CD45RO^+^ TILs, although the survival advantage associated with high TIL densities was independent from these molecular parameters.

### 1.7. TIL Density in Relation to Tumor Pathological and Molecular Features

Two issues were unclear at the beginning of this journey. First, the relative contribution of MSI colon cancers to the pool of tumors with high TILs was not initially considered. Second, a complete stage independency of TILs as a prognostic factor was initially claimed, to be later progressively refined. The intersection between MSI and high TIL amounts was well known to researchers dealing with MMR defects. This overlap was well portrayed by those who pointed out that the peculiar genetic instability of MMR deficient tumors ensues in the continuous and massive generation of frameshifted sequences that leads to the production of abnormal peptides acting as neoantigens [53]. These tumor antigens triggered a massive immune response mediated by high infiltration of cytotoxic immune cells which could exert antitumor activity slowing the ability of the tumor itself to spread [31,32,53]. These pioneer studies reported that patients whose colon cancer showed both MSI-high and high intraepithelial lymphocytes (assessed as CD3^+^, CD8^+^, and GrB^+^ cells) had a significantly reduced risk of death as compared to patients having only one of the two features. Namely, patients showing high GrB^+^ lymphocytes and MSI phenotype were those showing the more favorable relapse-free rates [27]. Other researches [54] pointed to the association of high load of TILs (assessed as intraepithelial CD8^+^ cells) with better long-term outcome of colon cancer patients. Furthermore, in light of the temporal pattern of the reduced risk associated with high CD8^+^ TILs (that became significant only after 2 years), they hypothesized an immune surveillance towards micro-metastases strongly correlated with the local amount of CD8^+^ cells. Notably, in the same work, Chiba et al. anticipated the relevant issue relative to the inverse correlation between the number of CD8^+^ cells and the colon cancer stage at diagnosis. In their series they reported a trend towards better outcomes of the colon cancers with MSI phenotype, yet they found no association between intraepithelial CD8^+^ TILs and MMR defects. Several papers contributed data that helped clarify these issues. We addressed the positive prognostic power of high CD3^+^ TIL densities, independent from the fraction of MSI tumors, in stage II colon cancer [14]. Therein, we showed that high TIL densities have protective power against the development of metachronous metastases, while such power was lost in stage III with a statistically significant interaction between the stage and CD3^+^ cells as to patient outcome, although we did not consider chemotherapy. A similar behavior was shown for tertiary lymphoid tissues, their density being related to that of CD3^+^ TILs and the high density of both were associated with better outcomes in stage II patients [55]. The densities of CD3^+^ TILs and of tertiary lymphoid tissue were significantly lower in patients with N0 colon cancer who relapsed than in those who did not, while such difference was not detected among node-positive patients, and the two responses were coordinated only in patients who did not relapse after surgery. Later, we were unable to detect any significant association of CD3^+^ TILs with the outcome in a cohort of patients with node-positive colon cancer receiving adjuvant therapy, while CD68^+^ cells (i.e., tumor associated macrophages [56]) were associated with significant predictive value for the responsiveness to 5-fluorouracil [57]. Differently from other authors who did not detect the relation between survival and the density of intraepithelial FOXP3 TILs [58], we assessed their density at the invasion front and found that increasing loads of FOXP3^+^ cells were associated with better outcomes. In addition, when coupled to the measurement of the density of CD3^+^ cells, the composite score can identify patients with MSS colon cancer who experienced the best survival, that is patients who had high densities of both populations had a better survival rate than patients with MSI colon cancer [59]. We were anyway unable to detect differences in the outcome of stage III according to the densities of CD3^+^ and FOXP3^+^ TILs in our cohort.

It progressively emerged that although MSI colon cancers contribute to the overall prognostic value of TILs, high densities of TILs also remain a significant prognostic factor in patients with MSS colon cancers. A collateral question may emerge, that is whether we should separately look for different cut-off values of TIL infiltration in MSI and MSS colon cancer, or simply let MSI colon cancers act on their own, due to their different behavior, despite the maintained prognostic value of TILs in MSI tumors [40]. However, it should be considered whether computing a unique TIL score could lead to a spuriously high TIL cut-off value for better outcomes, due to inherent higher loads of TILs in MSI colon cancers. In our institutional cohort comprising 654 patients with stage II and III colon cancers, assessed for CD3^+^ TIL density at the tumor invasive front and defined by their MS-status, in 87 MSI colon cancers (15% of the series) we computed a cut-off value close to the 4th quartile density (Youden’s index = 7.6% of CD3^+^ TIL immune-reactive area, at ROC curve analysis, sensitivity = 0.94, specificity = 0.32, and AUC = 0.59), and statistical significance of the logistic model had a *p*-value equal to 0.21 (OR 0.93; 95% CI 0.82–1.04). On the other hand, taking into account MSS colon cancers (*n* = 567), the cut-off value laid in the middle of the 3rd quartile and had lower CD3^+^ TIL value (Youden’s index = 3.3 of CD3^+^ TIL immune-reactive area, at ROC curve analysis sensitivity = 0.76, specificity = 0.36, and AUC = 0.57; logistic model OR 0.91; 95% CI 0.85–0.97, *p* = 0.004).

Interestingly, one additional subgroup of colon cancers with increased (intraepithelial) CD8^+^ TILs comprises those with pathogenic somatic mutations of *POLE* [39]. As emerged from a pooled analysis of patients from clinical trials (comprising QUASAR2 and PETACC3), these patients (66 out of 6517, 1%) with immunogenic colon cancers had an excellent prognosis, even better than those with MSI colon cancers, particularly in stage II. Due to the study design, it is not possible to establish whether such association was independent from adjuvant treatment. However, a recent paper confirmed the association of colon cancer harboring somatic mutations of the exonuclease domain of *POLE* with infiltrating CD8^+^ cells and excellent outcome [60].

The second issue concerns the inverse relationship between TIL loads and the tumor stage, which remains largely unsolved. It is unclear whether lower TIL loads observed in advanced stage represent [54] the fate of poorly immunogenic tumors originating with low TIL amounts, or whether immune evasion could take place in a fraction of cases leading to a reduction of the number of TILs over time. The latter explanation may also be indirectly supported by recent data which show that the protective value of high TIL densities within stage III decreases with the advancement of the components of the TNM staging system (that is high- vs low-risk stage III tumors) [42]. In a study including 779 colon cancers of all stages and taking into account MS and *BRAF* status, Wirta et al. [61] found that IS composed of CD3^+^ and CD8^+^ TILs was not superior to TNM staging as an independent prognostic factor, yet it could discriminate patients survival within each stage, particularly in stage IIa.

The large international validation of the IS, across stages I to III, according to two (high vs. low) or three (high-intermediate-low) levels, reinforced the notion of the prognostic value of TILs in stage II prognostication, independent from clinical-pathological variables, and also from the contribution of MSI colon cancers [46]. With respect to the latter, it appeared that the IS could also differentiate the outcome within this molecular subset. The capability of the IS to predict patient overall survival (OS) was superior to any other parameter individually considered (including T and N components). On the other hand, IS predictive ability was inferior to clinical parameters altogether. Nevertheless, when IS was added to the global model, the overall performance was improved. In this study, stage III patients were one third of all patients included, and adjuvant treatment was not considered among the covariates. One study by Emile et al. [62] assessed in a historically prospective way the predictive role of CD3^+^ TILs and their distance from the invasive margin in 1031 stage III patients from the PETACC8 study, who received adjuvant therapy. Patients with a high score of immune response experienced a better time to recurrence (14.4% vs. 21.1% at two years; *p* = 0.02) and a longer disease-free survival (DFS) than those with a low score (81% vs. 72% at three years [HR 0.69, 95% CI 0.51–0–92]; *p* = 0.01). As to molecular tumor features, the significant survival advantage associated with a high immune response score was maintained in patients with MSS but not in those with MSI colon cancer, and in those with wild-type *RAS*/*BRAF* but not in those with mutated colon cancers.

One other study performed on colon cancer patients from the PETACC8 trial (*n* = 1018), assessed the predictive value of CD3^+^ and CD8^+^ TILs at the invasive margin and the tumor core [24]. Using a LASSO algorithm, Reichling et al. derived what they called the DiGital tuMor pArameTErs, or DGMate score, based on 8 digital variables (out of 127 different parameters) associated to the outcome of patients. In this study, higher CD3^+^ cells at the tumor core/invasive margin and CD8^+^ cells at the core were associated with a better relapse-free survival, although CD3^+^ cells at the tumor core by themselves performed like CD3-CD8-based IS. This study also reported that together with CD3^+^ TILs, the stromal area of the invasive margin plus digital parameters has a better predictive value than the IS-like approach. In this analysis, the immune parameters were predictive in stage III, but with different performances in stage IIIa and IIIb, leading to propose a nomogram-like predictor in which immuno-digital scores contribute to the estimation of patient outcome together with the variation of T, N, and the *RAS* status.

One other study analyzed the densities of intra-tumoral CD3^+^ and CD8^+^ in TMA from 1804 colon cancers from QUASAR2 and VICTOR trials and reported that CD8^+^ TILs were a better predictor than the CD3^+^ ones [63]. Based on multivariable analysis, the risk reduction associated with increasing densities of CD8^+^ TILs was independent from MMR defects, *POLE* mutation and chromosomal instability. Interestingly, such reduction was uneven across strata defined by T and N parameters. It was evident in high risk (defined as pT4, N1–2; HR 0.87; 95% CI 0.79–0.97; *p* < 0.001), absent in low risk (pT3N0; HR 1.3, 95% CI 0.87–1.21), and only modest in intermediate risk (pT4N0 or PT1–2, N1–2; HR 0.92, 95% CI 0.86–1; *p* = 0.046). As the patients received adjuvant treatment, the results concerning patients with pT3N0 colon cancer may not be surprising, in analogy with the lack of protective effects by other TIL subpopulations in these patients [59], yet the results in stage III differ from those reported by other studies.

### 1.8. Latest Research on TILs and Adjuvant-Treatment in Stage III CRC

Two more papers published in 2020 addressed the predictive role of lymphocytes in stage III colon cancer. The one by Pages shows that high (intermediate plus high) amounts of TILs, assessed according to the IS methodology, are associated with better outcomes of patients with stage III colon cancer receiving FOLFOX [41]. In this study including 1322 patients from the IDEA France (multi center, two-arm, open-label, randomized phase III trial comparing 3 vs. 6 months adjuvant fluoropyrimidine and oxaliplatin-based adjuvant therapy), the survival benefit of having intermediate-high (i.e., above the first quartile) TIL loads was evident across stage III (3 year DFS, 77.1% for intermediate-high, vs. 66.8% for low TILs), as well as according to categorization in 3 (low-intermediate-high) or 5 (I0 to I4) groups according to TIL densities. However, the survival benefit conferred by intermediate-high TILs was more pronounced in patients with low-risk (i.e., T1–T3, N1) cancers than in those with high-risk (i.e., T4 and/or N2) cancers. Thus, adding a T/N stage improved the discrimination capacity of the model; for high-risk patients the IS did not achieve significant discrimination. Accordingly, the clinical behavior of high-intermediate densities of TILs as compared to low ones also differed according to the class of risk determined by the TNM, indicating that the T and N parameters modify the predictive ability of TILs in stage III patients undergoing adjuvant therapy. Considering the time frame of FOLFOX treatment as purposed by the IDEA study, an intermediate-high IS score was associated with a better response to 6-month treatment than to a 3-month treatment, irrespective of the risk class, as determined by the TNM parameters. In light of the reduction of the predictive power of TIL densities in high-risk colon cancer, this result seems to support a residual ability of high TIL densities to promote a better response to adjuvant therapy if sustained over time.

The other paper by Galon and the collaborators of an international consortium was aimed to evaluate the consensus IS in 763 stage III colon cancers from retrospective cohorts of patients treated with FU-based adjuvant therapies (spanning FOLFOX, XELOX, and FOLFIRI) from different continents [42]. Therein the IS was again evaluated considering several density intervals (that is 2, 3, and 5 different levels), and results again showed that patients with TIL densities above the first quartile showed a better survival than those with less TILs. Overall, recurrence rates at 5 years were 30.6%, 36.8%, and 50.4% for high, intermediate, and low IS, respectively, on a 3 levels categorization. As to MS-status, which was determined in 65% of the patients, subtracting MSI cases did not alter the predictive meaning of the IS. With respect to low and high-risk classes, the IS significantly predicted survival in subgroups of stage III colon cancer. Applying multivariable analysis, IS, T and N were the most important parameters for predicting OS. Accordingly, it remains unclear whether IS could be effectively considered independent of the T and N staging factors.

## 2. Current Status of Treatment for Stage II and III Colon Cancer, and Hints for Refinement from Translational Studies

### 2.1. Current Standards for Adjuvant Therapy

Since the mid-2000s, six months of either FOLFOX or XELOX became the standard adjuvant treatment regimen for stage III colon cancer, succeeding the positive results of the MOSAIC (Multicenter International Study of Oxaliplatin/5FU-LV in the Adjuvant Treatment of Colon Cancer) trial [64,65], which improved adjuvant standard treatment from FU/leucovorin or capecitabine to FOLFOX for stage III colon cancer patients. In an updated analysis [65], the 6-year OS rates were 78.5% and 76% in FOLFOX4 and FU/leucovorin, respectively (HR 0.84; 95% CI, 0.71–1.00; *p* = 0.046). The 6-year OS rates in the stage III disease were 72.9% and 68.7%, respectively (HR 0.80; 95% CI, 0.65–0.97; *p* = 0.023) (2). The advantage of oxaliplatin in combination with capecitabine in the adjuvant setting was later established in a study that compared capecitabine with bolus FU/leucovorin [66].

The main challenge of FOLFOX or XELOX in clinical practice is oxaliplatin dose-related neuropathy [64,65,66,67], which alters the quality of life in nearly all patients during therapy and in many for a long time after treatment end. Recently, the IDEA (International Duration Evaluation of Adjuvant chemotherapy) prospective, preplanned analysis accumulated data from six large randomized trials worldwide (i.e., SCOT, TOSCA, IDEA France [GERCOR/PRODIGE], Alliance/SWOG 80702, ACHIEVE, and HORG) to evaluate the non-inferiority of 3 months of adjuvant FOLFOX/XELOX compared with the standard 6 months treatment for patients with stage III colon cancer [68]. Grade 3 or greater neurotoxicity was significantly higher in the 6-month arm compared with the 3-month arm (16% vs. 3% with FOLFOX, 9% vs. 3% with XELOX; both *p* < 0.0001). The disease-free survival (DFS) non-inferiority of 3 months with oxaliplatin-based adjuvant therapy, the primary endpoint of the analysis, could not be confirmed overall in stage III colon cancer. However, the 3-months versus 6-month DFS HR was 1.01 (95% CI, 0.90–1.12) in T1-3 N1 disease and 1.12 (95% CI, 1.03–1.23) in T4 or N2 diseases, indicating that the regimen choice (FOLFOX or XELOX) and risk group (T1-3N1 and T4 or N2) should be considered in evaluating the non-inferiority of 3 months of oxaliplatin-based adjuvant therapy [68,69]. While data from randomized clinical studies have recognized clear survival advantages of adjuvant chemotherapy in stage III colon cancer patients [68,70], adjuvant therapy may not add enough benefit for cancer that invades the muscularis propria but has no lymph node involvement (stage IIA), while adjuvant chemotherapy might even be detrimental to patients with stage II colon cancer and mismatch repair (MMR) deficiency [7,30,71]. Nevertheless, it is well known that stage II tumors that penetrate the surface of the visceral peritoneum (T4a) and that directly invade or adhere to other organs or structures (T4b) may have a worse prognosis compared with patients with T3N1 disease. Therefore, the agreement of oncologists may not fulfill undisputed standards when they are challenged by decision in certain clinical settings. Accordingly, the discussion with patients about pros and cons of adjuvant chemotherapy remains part of the operative decision making in the management of high-risk stage II colon cancer, as defined by the one of followings: T4 lesions, bowel obstruction or perforation, less than 12 lymph nodes in the surgical specimen, and poorly differentiated histology [72]. Large vessel invasion, perineural and extramural vascular invasion also are considered high-risk features of recurrence [73]. Along the same line, inconsistencies exist in the definition of high-risk stage II disease, as reported in Table 1. The current lack of standardization in risk stratification diminishes the informative confidence of each feature. Negative prognosis has not been steadily associated with benefit from adjuvant chemotherapy, as underlined by a SEER (surveillance, epidemiology, and end results) Medicare database study in which patients with stage II colon cancer receiving adjuvant therapy with any high-risk feature did not have significant survival advantage as compared to patients with any high-risk factor treated with surgery alone [74]. Nevertheless, widespread international guidelines [75,76,77] advocate adjuvant FU/capecitabine with or without oxaliplatin for high-risk stage II disease. This recommendation is largely based on an exploratory analysis of the MOSAIC trial which showed an absolute 7% increase in the probability of DFS at 5 years with the oxaliplatin-containing regimen, though it did not reach statistical significance [67,78].

Pooled analyses have shown a limited 2% to 4% benefit in 5-year DFS for FU-based adjuvant therapy in stage II colon cancer [80,81]. These results were confirmed in the QUASAR (quick and simple and reliable) study (5-year OS, 80.3% for chemotherapy, 77.4% for observation; HR, 0.83; *p* = 0.02) [82], which, coupled with the results from the MOSAIC trial, prove no significant benefit for the addition of oxaliplatin to FU/leucovorin in unselected stage II disease [65] or even high-risk patients with stage II disease [83], and support single-agent fluoropyrimidine-based therapy as the favored treatment for a patient with stage II disease in whom chemotherapy is considered suitable.

Adjuvant therapy is prescribed in the light of an increased risk of disease progression, and equally it is assumed that a percentage of patients who receive adjuvant therapy are cured by primary surgery alone. At present, the classification by anatomic TNM remains the only validated prognostic tool in the adjuvant setting, and clinical practice is accordingly centered on T and N staging. As an example, also consider that the MSI/MMR-deficient phenotype is relevant for decision making within this frame [6,7]. The American Joint Committee of Cancer revises the staging systems each 6 years in accordance with novel findings from clinical research and epidemiologic data [84]. Real-life data on more than 200,000 stage III colon cancers showed that no more than 60% of patients receive adjuvant chemotherapy [85,86]. Comparatively, only 20% of patients with stage III colon cancer would benefit from adjuvant therapy, 80% of patients being exposed to risks and toxicity of multi-drug chemotherapy [85,86].

### 2.2. MS-Status in Support to TNM Staging System in Adjuvant Treatment Management

MSI/dMMR has long been established as a positive prognostic marker in the adjuvant setting [7,71,87]. It is also applied for therapeutic decisions since adjuvant chemotherapy with fluoropyrimidine alone is not indicated for patients with stage II dMMR colon cancer, given their favorable stage-adjusted survival and low recurrence rates [87,88]. Numerous assumptions have been suggested to provide biologic explanation by which FU-based adjuvant chemotherapy does not improve survival in dMMR colon cancer patients. Among others, it is the immune response characterized by the distinctive lymphocytic infiltrates of dMMR tumors [89], which could be counteracted by immunosuppressive effects associated with chemotherapy. For instance, in vitro studies have shown dissimilar efficacy of FU between dMMR and pMMR tumors [90,91]. In contrast to stage II, the MSI/dMMR phenotype have less prognostic value in stage III colon cancer and the combination of a fluoropyrimidine and oxaliplatin for 3–6 months is recommended [6,7,64,87,92,93,94] regardless of MMR status, although in one study the prognostic impact of MMR value was dependent on the primary tumor site, with a statistically significant DFS advantage in proximal and N1 tumors [94]. This effect is seemingly unrelated to the chemotherapy regimen used, but likely correlated to intrinsic biologic tumor features.

### 2.3. Biomarkers for Refining Patient Selection for Adjuvant Therapy

The potential for further refinement in patient selection for adjuvant chemotherapy in stage III colon cancer has been suggested by reports from the PETACC-3 biomarker cohort where a recursive partitioning analysis identified a tumor subgroup with dMMR and intact SMAD4 expression that had survival rates analogous to stage II disease [92,95]. MSI tumors are usually characterized by higher levels of a specific subset of infiltrating lymphocytes (CD8^+^, CD4^+^) [21,48,96]. Also, lymph node count at surgery has been associated with lymphocytic reaction [97,98,99,100,101], which is positively related to MSI and improved prognosis [21]. Remarkably, gene expression signatures that reveal stromal infiltration and epithelial–mesenchymal transition [37,102] or carcinoma-associated fibroblasts [103,104] may be associated with variable immune infiltrates in the tumor microenvironment [46,105], and seem related to the resistance to standard adjuvant therapy [106,107], reinforcing the awareness that the immune microenvironment remains a relevant determinant of the hazard of distant spreading. Accordingly, interesting prognostic models have been obtained by combining multiple gene expression data with established clinicopathological features [108]. Microarray-based versions of risk scores applied to a large independent cohort of 688 stage II/III tumors provided useful prognostic information, although ultimately they could only marginally improve the models based on recognized risk features [108].

Extensive evidence supports the use of predictive in situ immune-cell infiltrate [28,29,30,32,33,34,50,67,103,104,105], but chemotherapy adds a complicating cofactor, as it affects the immune system not solely negatively but it may as well exert immunostimulatory properties. It should be considered whether the clinical efficacy of conventional anticancer drugs also derives from their capability to prompt specific immune response, such as immunogenic cell death [109,110,111]. For example, high densities of tumor-associated macrophages, specifically in metastatic lymph-nodes, may differentiate stage III colon cancers benefitting from 5-fluorouracil adjuvant therapy [57]. The advantage of chemotherapy in the context related to pre-existing immunity has been recently proposed by the International Society for Immunotherapy of Cancer—IS consortium, which conducted a large retrospective analysis aimed at validating the predefined consensus IS for patients with stage III colon cancer [42]. The consensus IS has been shown to accurately stratify high- and low-risk colon cancers with significant differences in survival rates. Both time to recurrence (TTR) and OS have been strongly correlated with local adaptive immune reaction [12,22,40] at the center of the tumor (CT) and at the invasive margin (IM) [112]. The Multicenter International Society for Immunotherapy of Cancer studied the Consensus IS for the prediction of survival and response to chemotherapy in stage III colon cancer [42]. Independently of age, IS categories defined separately of clinical data [46], predicted clinical outcome. Specifically, low-IS stage III colon cancers did not benefit from chemotherapy, while stage III colon cancer with an elevated preexisting immunity had a higher chance to benefit from chemotherapy, even in a low-risk stage. This indicates that an active chemotherapy regimen may partially depend on the existence of elevated densities of tumor infiltrating T cells. However, though the number of cases was limited, none of the highest IS (I4) colon cancers relapsed, even when treated with surgery alone, reinforcing the assumption that these cases could be spared from chemotherapy. The MSI status, usually applied for treatment evaluation, seemed to be dependent on the IS, as confirmed by multivariable stratified Cox analyses. Also, *RAS* and *BRAF* mutational status were apparently not related to survival. Limits of this study are correlated to the heterogeneity of the analyzed cohort, with MSI and mutational status available only in a subset of patients. The authors concluded about the importance of improving colon cancer classification by including the consensus IS in cancer guidelines (i.e., National Comprehensive Cancer Network, AJCC/UICC-TNM, College of American Pathologists), as the fifth edition of the WHO classification has just been completed. Certainly, both the IS and genetic markers should be explored in larger prospective studies to establish whether mutations arising at various disease stages have a distinctive effect on the adaptive immune infiltrates and on clinical outcome. Though risk stratifying stage III colon cancer is likewise achievable with these prognostic features, a superior level of evidence is necessary before a recommendation to avoid chemotherapy in the low-risk subgroup can be generated [113,114]. Accordingly, acknowledging the huge amount of data generated so far, the picture concerning stage III colon cancers requires additional evidence to understand in what set of patients TILs are more informative in guiding post-surgical management.

## Figures and Tables

**Table 1:** Definitions of high-risk stage II colon cancer according to international guidelines.

Guidelines	Definition
European Society for Medical Oncology [75]	pT4 stage and/or <12 lymph nodes assessed or multiple intermediate risk factors (i.e., lymphatic, perineural or vascular invasion, tumor obstruction, histological grade 3, preoperative CEA > 5 ng/mL) MSI/MMR status
American Society of Clinical Oncology [76]	extramural vascular invasion, grade 3/poorly differentiated tumors, T4 stage/perforation, obstructive tumors, mucinous tumors, examination of less than 12 lymph nodes, and tumor budding
National Comprehensive Cancer Network [79]	pT4 tumor, lymphovascular or perineural invasion, localized perforation or bowel obstruction, close, indetermined or positive margins, poorly differentiated tumor (excluding tumors that are MSI-H), or lymph node sampling <12 nodes
